# Poor pregnancy outcome after octreotide treatment during pregnancy for familial hyperinsulinemic hypoglycemia: a case report

**DOI:** 10.1186/1756-0500-7-804

**Published:** 2014-11-17

**Authors:** Gitte O Skajaa, Elisabeth R Mathiesen, Elisabeth Iyore, Henning Beck-Nielsen, Espen Jimenez–Solem, Peter Damm

**Affiliations:** Center for Pregnant Women with Diabetes, Copenhagen University Hospital, Copenhagen, Denmark; Departments of Obstetrics, Copenhagen University Hospital, Copenhagen, Denmark; Departments of Endocrinology, Copenhagen University Hospital, Copenhagen, Denmark; Departments of Neonatology, The Juliane Marie Centre, Copenhagen University Hospital, Copenhagen, Denmark; The Institute of Clinical Medicine, Faculty of Health and Medical Sciences, University of Copenhagen, Copenhagen, Denmark; Department of Endocrinology, Odense University Hospital, Odense, Denmark; Department of Clinical Pharmacology, Bispebjerg Hospital, Copenhagen, Denmark

**Keywords:** Pregnancy, Diabetes, Hyperinsulinemia, Octreotide, Necrotizing enterocolitis, Intrauterine growth retardation and pregnancy outcome

## Abstract

**Background:**

Late familial hyperinsulinemic hypoglycemia is characterized by recurrent episodes of hypoglycemia and an inappropriate insulinemic response. Treatment with octreotide (somatostatin analogue) reduces the prevalence of clinical significant hypoglycemia and might be beneficial during pregnancy. To our knowledge this is the first report of a woman with late familial hyperinsulinemic hypoglycemia experiencing pregnancies with and without octreotide treatment.

**Case presentation:**

A 35-year-old Caucasian woman known to suffer from late familial hyperinsulinemic hypoglycemia due to a well-known mutation in the insulin receptor gene has been pregnant 6 times. The patient was treated with injections of Sandostatin LAR® (octreotide) during the first four pregnancies. Her first pregnancy in 1999 was unknown until approximately 25th gestational weeks with fatal intrauterine growth retardation. The following two pregnancies were terminated on parental request after a chorion villus biopsy revealed the mutation causing late familial hyperinsulinemic hypoglycemia. During the fourth pregnancy, in which the fetus also had the mutation, serial ultrasound examinations showed a small fetus with appropriate growth. At birth the girl was small for gestational age. She was admitted to the neonatal special care unit due to low blood glucose and intravenous glucose and early feeding was initiated. One day old, her condition deteriorated with signs of an abdominal catastrophe indicating necrotizing enterocolitis. After two laparotomies – both confirming necrotizing enterocolitis - the child died 8 days after birth.

In the following two pregnancies Sandostatin LAR® was stopped before pregnancy and the patient was treated only with diet restriction and intensive glucose monitoring. Both pregnancies ended successfully. One child carried the mutation and was small for gestational age at birth while the other child did not carry the mutation and had normal birth weight.

**Conclusion:**

In a woman with late familial hyperinsulinemic hypoglycemia octreotide was given during the first four pregnancies resulting in 2 cases of early termination of pregnancy on parental request and 2 cases of inappropriate fetal growth and unviable outcome. The following two pregnancies treated with diet only had a successful outcome.

## Background

Late familial hyperinsulinemic hypoglycemia (FHH) is characterized by an inappropriate insulinemic response [1, 2]. Some of the causes can be gain-of-function mutations in glucokinase or glutamate dehydrogenase, abnormal pyruvate-induced insulin release, insulinomas or, as in the present case, a missense mutation in the insulin receptor gene.

This rare missense mutation in a tyrosine kinase domain in the insulin receptor causes severe postprandial hypoglycemia, insulin resistance and decreased insulin clearance. The mutation is autosomal-dominant and with variable expression [1].

One way to treat recurrent episodes of severe hypoglycemia due to hyperinsulinemia is injections with octreotide. This is a synthetic but more potent version of the natural hormone somatostatin, which inhibits insulin, glucagon and growth hormone with a prolonged duration of action. Most of the limited experience with administration of octreotide during pregnancy comes from women being treated for acromegaly, and treatment with octreotide during pregnancy in these cases is generally considered safe [3].

## Case presentation

A 35-year-old Caucasian woman known to suffer from late FHH due to a well-known mutation [1] has been pregnant 6 times with the result of 2 surviving children. All the pregnancies are summarized in Table 1.

**Table 1:** **Clinical characteristics of the patient’s pregnancies**

Year	Mutation (yes/no)	Gestational age (week+day)	Birth weight (g)	Octreotide during pregnancy (yes/no)
1999	Unknown	25	Unknown	Yes
2008	Yes	10 + 3	Termination	Yes
2008	Yes	10 + 6	Termination	Yes
2009	Yes	37 + 6	2477	Yes
2010	Yes	36 + 0	2246	No
2012	No	37 + 2	2873	No

The disease’ first manifestation was recurrent episodes of convulsions with loss of consciousness at the age of 12. This was interpreted and treated as epilepsy until the age of 21 (year 1999). At this time, measurements of capillary blood glucose levels in the range of 1.1 – 1.5 mmol/L during seizures led to measuring fasting plasma insulin (341 pmol/L) and S-insulin-to-C-peptide ratio (0.5). The patient began treatment with 30 mg intramuscular injections of Sandostatin LAR® (octreotide) every four weeks and new neuroglycopenic attacks were avoided. Three generations of family members showed similar episodes of severe hypoglycemia and a genetic study of the family determined the genetic cause [1].

Her first pregnancy in 1999 was unacknowledged until approximately 25th gestational week and showed fatal intrauterine growth retardation. She had received Sandostatin LAR® (30 mg every four weeks) throughout this pregnancy.

In 2008 she was pregnant twice but each time a genetic analysis from a chorion villus biopsy revealed the mutation causing late FHH. The couple chose an induced abortion in both cases.

In contrast, the pregnancy in 2009 was continued although the fetus had the mutation. Throughout pregnancy the patient was treated with 30 mg Sandostatin LAR® every four weeks until gestational age (GA) 30 weeks. Serial ultrasound examinations during pregnancy showed a small fetus with appropriate growth velocity. At birth the infant was small for gestational age (SGA) with a birth weight of 2477 g, corresponding to -1.74 standard deviations (SD) below the population mean [4]. Gestational diabetes was diagnosed at GA 27 weeks and her blood glucose was well controlled on diet alone. One and a half hours after an uncomplicated labor and delivery at GA 37 weeks +6 days, the newborn girl was admitted to the neonatal special care unit due to low blood glucose and IV glucose and early feeding was initiated. One and half days old, her condition deteriorated with signs of an abdominal catastrophe indicating necrotizing enterocolitis (NEC). Laparotomy with resection of necrotic colon and a colostomy was performed and treatment with IV antibiotics initiated. After the laparotomy the child briefly tolerated feeding but few days later the colostomy became necrotic and a second laparotomy showed intestinal necrosis from ventricle to rectum. The child died 8.5 days old.

Based on these poor pregnancy outcomes and casuistic reports on NEC in newborns treated with octreotide [5, 6] it was decided to stop Sandostatin LAR® before and during following pregnancies. The patient was treated with diet and daily self monitored plasma glucose with intermittent continuous glucose monitoring with alarms set for hypoglycemia (Guardian® Real-time Continuous Glucose Monitoring System; Medtronic Minimed). The diet consisted of at least 6–8 meals daily with carbohydrates of low-glycemic index.

In the following pregnancy in 2010 the female fetus had the mutation and serial ultrasound measurements documented a small fetus with appropriate growth velocity. The patient followed the described plan with diet and glucose monitoring and no episodes of severe hypoglycemia occurred. Birth weight at GA 36 weeks +0 days was 2246 g (SGA and corresponding to -1.56 SD). The final pregnancy in 2012 the same treatment regime was followed and resulted in a healthy girl without the mutation. Birth weight at GA 37 weeks +0 days was 2873 g (-0.25 SD). Both infants had a spontaneous vaginal delivery and an uneventful neonatal period.

## Conclusions

We here present 6 pregnancies in the same woman carrying a rare mutation causing late familiar hyperinsulinemic hypoglycemia. Four pregnancies were continued beyond the first trimester and 3 of these resulted in a small for gestational age infant. Mutation status is not known for the first of the 6 pregnancies, the 2^nd^ to 5^th^ had the mutation, while the last child did not have the mutation.

Theoretically the presence of a mutation causing late FHH might cause inappropriate fetal growth. Glucose passes the placenta and facilitates an increase in the insulin level of the fetus. It is well known that insulin has the effect of stimulating fetal growth [7]. A fetus with the mutation in the insulin receptor could be immune to the direct effect of insulin as well as the indirect effect via decreased uptake of metabolites such as glucose, lipids and amino acids. This might cause impaired growth. In contrast, a fetus without the mutation will have an appropriate insulin response that facilitates growth leading to an appropriate birth weight. This is in accordance with the studies from the group of Hattersley et al. looking into the effect of fetal glucokinase mutations in offspring of women with Maturity Onset Diabetes of the Young 2 [8].

Data on safety of octreotide application during pregnancy are very scarce and considering our report of two fatal pregnancies during octreotide treatment one must consider whether it is always safe to administer octreotide during pregnancy.

There are no reports on NEC in infants being exposed to octreotide during pregnancy, but Laje et al. reported 8 cases of NEC in infants with severe hypoglycemia secondary to hyperinsulinemia being treated with octreotide [9]. On this background octreotide was not used in the last 2 pregnancies. One of these 2 pregnancies where octreotide was not used resulted in a small for gestational infant (+FHH mutation) while the other resulted in an appropriate weight infant (-FHH mutation).

The combination of a mutation causing impaired fetal growth with a drug that could affect fetal growth should also be considered as a possible culprit. This could explain why treatment with octreotide during pregnancy in women with acromegaly is considered safe [10]. Also it could explain why the pregnancy in 2010 with the mutation present and no octreotide treatment ended successful; although resulting in a small for gestational age infant.

In this case report we are not able to draw a final conclusion concerning causality.

However, we believe that our case supports the notion that a mutation causing decreased fetal insulin function might cause fetal impaired growth. Furthermore, the potential role of octreotide in the development of NEC and poor outcome in prenatally exposed infants should also be considered and investigated. Until then, octreotide treatment during pregnancy must be well indicated and alternative treatments thought of before choosing this course of treatment. Caution must be taken if the patient in addition has a mutation that might induce impaired fetal growth on its own.

## Consent

Written informed consent was obtained from the patient for publication of this Case Report and any accompanying images. A copy of the written consent is available for review by the Editor-in-Chief of this journal.

## Abbreviations

IV: Intravenous
FHH: Familiar hyperinsulinemic hypoglycemia
GA: Gestational age
NEC: Necrotizing enterocolitis
SD: Standard deviation
SGA: Small for gestational age.
